# Role of Traditional Risk Factors and Antiretroviral Drugs in the Incidence of Chronic Kidney Disease, ANRS CO3 Aquitaine Cohort, France, 2004–2012

**DOI:** 10.1371/journal.pone.0066223

**Published:** 2013-06-12

**Authors:** Philippe Morlat, Alexandre Vivot, Marie-Anne Vandenhende, Frédéric-Antoine Dauchy, Julien Asselineau, Edouard Déti, Yann Gerard, Estibaliz Lazaro, Pierre Duffau, Didier Neau, Fabrice Bonnet, Geneviève Chêne, the Groupe D’epidémiologie Clinique du Sida en Aquitaine (Gecsa)

**Affiliations:** 1 Univ. Bordeaux, ISPED, Centre INSERM U897-Epidemiologie-Biostatistique, Bordeaux, France; 2 INSERM, ISPED, Centre INSERM U897-Epidemiologie-Biostatistique & CIC-EC7, Bordeaux, France; 3 CHU de Bordeaux, Pôle de Santé Publique, Clinical Epidemiology Unit (USMR), Bordeaux, France; 4 CHU de Bordeaux, Pôle de Santé Publique, Coordination Régionale pour la Lutte contre le VlH (COREVIH), Bordeaux, France; 5 CH de Dax, Service de Maladies Infectieuses, Dax, France; 6 Univ. Bordeaux, UFR des sciences médicales, Bordeaux, France; University Hospital Zurich, Switzerland

## Abstract

**Objective:**

To examine the role of antiretroviral drugs (ART), HIV-related and traditional risk factors on the incidence of chronic kidney disease (CKD) in HIV-infected patients.

**Design:**

Prospective hospital-based cohort of HIV-infected patients from 2004 to 2012.

**Methods:**

CKD was defined using MDRD equation as an estimated glomerular filtration rate (eGFR) less than 60 ml/mn/1.73 m^2^ at 2 consecutive measurements ≥3 months apart. Poisson regression models were used to study determinants of CKD either measured at baseline or updated. ART exposure was classified as ever or never. We additionally tested the role of tenofovir (TDF), whether or not prescribed concomitantly with a Protease Inhibitor (PI), taking into account the cumulative exposure to the drug.

**Results:**

4,350 patients (74% men) with baseline eGFR>60 ml/mn/1.73 m^2^ were followed for a median of 5.8 years. At the end of follow-up, 96% had received ART, one third of them (35%) jointly received TDF and a PI. Average incidence rate of CKD was 0.95% person-years of follow-up. Incidence of CKD was higher among women (IRR = 2.2), older patients (>60 y vs <45 y: IRR = 2.5 and 45–60 y: IRR = 1.7), those with diabetes (IRR = 1.9), high blood pressure (IRR = 1.5), hyperlipidemia (IRR = 1.5), AIDS stage (IRR = 1.4), low baseline eGFR (IRR = 15.8 for 60<eGFR<70 ml/mn/1.73 m^2^ vs >90 and IRR = 7.1 for 70<eGFR<80 ml/mn/1.73 m^2^), current CD4+<200 cells/mm^3^ vs >500/mm^3^ (IRR = 2.5), and exposure to TDF (IRR = 2.0). Exposure to TDF was even strongly associated with CKD when co-administered with PIs (IRR = 3.1 vs 1.3 when not, p<0,001). A higher risk of CKD was found when tenofovir exposure was >12 months [IRR = 3.0 with joint PIs vs 1.3 without (p<0.001)]. A vast majority of those developing CKD (76.6%) had a baseline eGFR between 60 and 80 ml/mn/1.73 m^2^.

**Conclusion:**

In patients with eGFR between 60 and 80 mL/min/1.73 m^2^, a thorough control of CKD risk factors is warranted. The use of TDF, especially when co-administered with PIs, should be mentioned as a relative contraindication in presence of at least one of these risk factors.

## Introduction

Chronic kidney disease (CKD) is defined by the National Kidney Foundation as evidence of either kidney damage or glomerular filtration rate (GFR) below 60 mL/min/1.73 m^2^ that persists for at least 3 months [1]. CKD is an important risk factor of cardio-vascular morbidity, hospitalizations and mortality, in the general population as in HIV-infected patients [2], [3]. Among untreated HIV-infected individuals, the occurrence of renal impairment is mainly due to HIV-associated nephropathy (HIVAN), although this was mainly described in black population [4]. Since the widespread availability of combination antiretroviral therapy (ART), HIVAN is rare but a longer survival of treated HIV-infected patients may expose them to other harmful factors for renal function. Therefore, in the ART era, the prevalence of CKD may reach 5 to 10% in several observational studies [5]–[8].

Traditional risk factors of CKD previously identified in the general population [9], [10] are frequently reported among HIV infected patients [6], [11], [12]. In addition, low CD4 count, high plasma HIV RNA or a history of AIDS-defining diagnosis, have been associated with a higher incidence of CKD [7], [11]–[13].

The role of antiretroviral drugs on renal function is beneficial on average but some specific drugs have nevertheless been identified as nephrotoxic. Nephrotoxicity of indinavir, tenofovir and more recently of boosted protease-inhibitors (PI/r) others than indinavir, have been reported [11], [14]. Moreover, concomitant exposure to PIs has been associated with a larger TDF exposure [15], a slower renal clearance of TDF [16] and a larger reduction of glomerular filtration rate [17]–[22]. Recently, Kalayjan et al reported a higher CKD risk when ART regimens contained tenofovir plus PI/r [23].

In a large ongoing clinic-based cohort of HIV-infected patients, we analysed the respective role of traditional versus HIV-related risk factors of CKD and the potential interaction between exposure to TDF and boosted PIs.

## Methods

Written informed consent was taken from all the study participants. The study was approved by the Ethics committee of Bordeaux University Hospital (Comité de protection des personnes).

The ANRS CO3 Aquitaine Cohort is a prospective hospital-based cohort of HIV-1 infected patients under routine clinical management, initiated in 1987 in the Bordeaux University Hospital and four other public hospitals in the Aquitaine region, South Western France. Inclusion criteria are: adult patients of the participating hospital wards with confirmed HIV-1 infection, having at least one follow-up after the first report, and having given informed consent. Visits occur usually every three months if the patient is treated, every six months otherwise. Detailed presentation of the cohort has been reported elsewhere [24].

### Estimation of Kidney Function

As creatinine was measured using a kinetic compensated Jaffe assay traceable to an Isotopic Dilution Mass Spectrometry (IDMS) determination, and as ethnicity was not registered in our cohort, glomerular filtration rate (eGFR) was estimated by the IDMS-traceable MDRD equation without ethnicity [25]:

eGFR = 175× (serum creatinine µmol/L×0.0113) −1.154×age −0.203×0.742 (if female).

CKD-Epi Equation was not used as it is validated only with enzymatic assays [26].

### Eligibility Criteria

Patients were included in this analysis if they had a follow-up visit between January 2004 and June 2012. Their follow-up was taken into account until December 2012. Patients were not included in this analysis if MDRD was not validated to estimate GFR (pregnant women, Body Mass Index (BMI) <18 kg/m^2^ or BMI >30 kg/m^2^, creatininemia <30 µmol/L, ascites) or when data were not sufficient enough to calculate MDRD formula, or if their first eGFR measure was <60 mL/min/1.73 m2 (i.e prevalent cases), or if they had less than two eGFR measures after a first normal measurement.

### Study Variables

Our main outcome was the incidence of chronic kidney disease (CKD), defined as two eGFR measures <60 ml/min/1.73 m^2^ three months apart.

Explanatory variables were either fixed or updated. Fixed variables included gender, HIV transmission group (injection drug user or other), initial eGFR (60–70, 70–80, 80–90 or >90 mL/min/1.73 m2) and age (<45, 45–60 or >60 years). Updated variables included BMI, AIDS stage, delay since HIV diagnosis, having reached the AIDS stage, Hepatitis C Virus (HCV) co-infection (defined as the detection of anti-HCV antibodies at least once at baseline or during follow-up), Hepatitis B Virus (HBV) co-infection (defined as the detection of HBV antigen at least once), history or presence of diabetes (defined by use of antidiabetic drugs, or fasting glycaemia >11 mmol/L or diagnosis reported by physician), history or presence of high blood pressure (defined by the use of antihypertensive agents or systolic blood pressure >140 mmHg or diastolic blood pressure >90 mmHg or diagnosis reported by physician), history or presence of hyperlipidemia (prescription of lipid lowering drugs, or fasting total plasma cholesterol >6.5 g/L or fasting triglyceridemia >2.2 g/L or diagnosis reported by physician), plasma HIV1-RNA (≥50 copies/mL), CD4 cell count and exposure to ART.

We computed the cumulative exposure to each class of ART since enrolment in the ANRS CO3 Aquitaine Cohort including nucleoside reverse transcriptase inhibitors (NRTI), non-nucleoside reverse transcriptase inhibitors (NNRTI), protease inhibitors (PI), and to each individual ART. We used dichotomous variables (ever vs never) to avoid biases due to the modification of drugs regimens according to the evolution of renal function assessment (i.e discontinuation of a specific drug when renal function deteriorates).

### Statistical Analyses

Person-years accrued from inclusion in this analysis (January 2004 or after) until the earliest of chronic kidney disease, loss to follow-up (LTFU) or close of the dataset (31 December 2012). LTFU was defined as not having attended the clinic since at least 24 months while being alive and without CKD at the last visit.

Incidence rate of CKD was calculated as the number of cases of CKD divided by the number of person-years of follow-up (PYFU). Cumulative probability of having CKD was estimated by the Kaplan-Meier method. Poisson regression models were used to study determinants of CKD whether measured at baseline or updated.

A multivariable final model was obtained by fitting a backward selection procedure from an initial model containing all variables with a p<0.25 in the univariable analyses. Statistical significance for selection was set at 0.05.

Tenofovir exposure was processed in different ways. In a first analysis, exposure to tenofovir was classified as ever/never as other ART-exposures. In order to investigate our hypothesis about increased nephrotoxicity of tenofovir when co-administrated with PI, two additional analyses were performed:

- firstly, tenofovir exposure was classified in four categories: never exposed, exposed less than 6 months, expose more than 6 months with joint exposure to PI during at least 6 months and ever exposed more than 6 months without joint exposure to PI during at least 6 months.
- secondly (and despite biases mentioned above), cumulative tenofovir exposure was classified in five categorical variables [0–6 months, 6–12 with or without concomitant (at least 6 months) exposure to PI and >12 with or without concomitant (at least 6 months) exposure to PI].

## Results

Between January 2004 and December 2012, 5,283 patients had at least one contact reported in the ANRS CO3 Aquitaine cohort. Among them, 933 were excluded from the analysis for the following reasons: estimation of eGFR by MDRD formula was not applicable for 412 patients (pregnant women, Body Mass Index (BMI) <18 kg/m2 or BMI >30 kg/m2, creatinine concentration <30 µmol/L, ascites, insufficient data to use and calculate MDRD formula), 260 patients had MDRD <60 mL/min and were considered prevalent cases of CKD and 261 patients had less than two eGFR measures after the first normal one. The main characteristics which differ (p<0.05) between included and non-included patients were: male gender (74.4% in included patients vs 63.8%), diabetes (8.0% vs 3.9%), hyperlipidemia (40.2% vs 36.3%), high blood pressure (19.5% vs 10.5%), HCV co-infection (26.6% vs 21.2%) and AIDS stage (24.4% vs 19.6%).

Baseline characteristics of the 4350 patients included in the analysis are shown in table 1.

**Table 1:** Patient’s characteristics at baseline and at end of follow-up, Aquitaine Cohort 2004–2012, N = 4350.

	Baseline			End of follow-up			
Variables	N	n	%	N	n	%	
Men	4350	3236	74.4				
Injection drug use	4350	701	16.1				
ART-naïve patients		1100	25.3	4350	184		4.2
Age (years)	4350			4350			
<45		2841	65.3		1755		40.3
45–60		1266	26.1		2095		48.2
>60		243	5.6		500		11.5
Creatinine Clearance (mL/min/1.73 m^2^)	4350			4350			
<60					289		6.6
60–70		396	9.1		248		5.7
70–80		768	17.7		543		12.5
80–90		968	22.3		789		18.1
>90		2218	51.0		2481		57
Diabetes*	4350	169	3.9	4350	252		5.8
Hyperlipidemia*	4350	1448	33.3	4350	2506		57.6
High blood pressure*	4350	392	9.0	4350	1187		27.3
Hbs Ag*	3752	207	5.5	4054	195		4.8
HCV Ab*	3717	788	21.2	4022	819		20.4
AIDS Stage	4350	855	19.7	4350	987		22.7
CD4 Lymphocytes/mm3	4174			4093			
<200		608	14.6		298		7.3
200–350		877	21.0		609		14.9
350–500		1013	24.3		935		22.9
>500		1676	40.2		2251		55
Plasma HIV RNA > 50 copies/mm3	4073	2337	57.4	4050	746		18.4
Exposure to tenofovir †	4350			4350			
Never		3215	73.9		1083		24.9
Less than 6 months		281	6.5		282		6.5
Ever with joint PI ≥ 6 months		241	5.5		892		20.5
Ever without joint PI ≥ 6 months		613	14.8		2093		48.2
	N	Median	IQR	N	Median		IQR
Cumulative exposure of NRTI †	3234	5.8	2.8–8.1	4147	8.6		4.3–13.0
Cumulative exposure of NNRTI †	1897	2.3	1.0–3.7	2652	3.0		1.3–6.3
Cumulative exposure of PI †	2138	3.0	1.2–2.3	3109	4.5		2.0–7.7
Cumulative exposure of tenofovir †	1136	1.1	0.5–1.8	3268	3.3		1.6–5.3

N: Number of available data.

IQR: Inter-quartile range; Ag: antigen; HCV: Hepatitis C Virus, Ab: Antibodies.

* See methods section for definition.

† Since enrolment in the ANRS CO3 Aquitaine Cohort (in years).

During a median duration of follow-up of 5.8 years (IQR 2.5–7.5–) accounting for 21983 PYFU, CKD occurred in 209 patients within a median time of 2.1 years (IQR 0.9–4.4). Incidence of CKD was 0.95 cases per 100 PYFU, 95% CI (0.83–1.09). Median annual eGFR decline in patients who progressed to CKD was 9.0 ml/mn/1.73 m^2^ [IQR: 3.8; 17.8].

**Figure 1:**
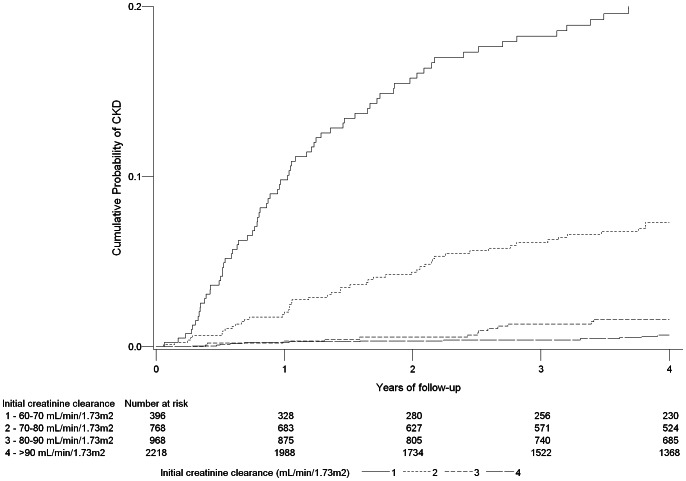
Cumulative probability of CKD according to baseline creatinine clearance in 4350 patients of the ANRS CO3 Aquitaine Cohort, 2004–2012.

Figure 1 shows that CKD occurred nearly always in patients with baseline eGFR<80 mL/min.

Six hundred and eighty four patients (15.7%) were LTFU.

One hundred and eighty seven (5.9%) patients without CKD died during follow-up and 22 (10.5%) among the 209 patients with CKD.

At the end of follow-up (table 1), 96% of patients had received ART (mean total cumulative exposure since enrolment in the ANRS CO3 Aquitaine Cohort: 9.1 years) among whom 35% jointly received tenofovir and a PI for at least 6 months any time during follow-up: atazanavir/r (33%), lopinavir/r (31%), fosamprenavir (13%), nelfinavir (12%) saquinavir (8%), others (3%).

Factors significantly associated (p<0.05) with a higher incidence of CKD in univariable analysis are shown in table 2. Among PIs, indinavir (Incidence Rate Ratio: IRR = 1.8; 95% CI [1.4–2.5]) and atazanavir (IRR = 1.4 [1.0–1.8]) were also significantly associated with CKD. Other PIs were not significantly associated although a trend was observed for lopinavir (IRR = 1.3 [1.0–1.7], p = 0.05).

**Table 2:** Factors associated with CKD in ANRS CO3 Aquitaine Cohort, N = 4350 patients and 209 cases.

		Univariable analysis			Multivariable analysis 1*			Multivariable analysis 2†		
Variables		IRR	95% CI	p-value	IRR	95% CI	p-value	IRR	95% CI	p-value
Age (years) :				<0.0001			<0.0001			<0.0001
	45–60 vs <45	2.5	1.8–3.4		1.7	1.1–2.4		1.7	1.2–2.6	
	>60 vs <45	5.9	4.0–8.7		2.5	1.6–4.0		2.6	1.6–4.1	
Women vs men		1.9	1.4–2.5	<0.0001	2.2	1.6–3.0	<0.0001	2.2	1.6–3.0	<0.0001
Injection drug use (yes vs no)		0.8	0.5–1.2	0.3						
Diabetes‡ (yes vs no)		3.2	2.1–4.7	<0.0001	1.9	1.2–3.0	0.008	1.9	1.2–3.0	0.006
Body mass index (for 1 more kg/m2 )		1.0	0.9–1.0	0.4						
High blood pressure‡ (yes vs no)		2.5	1.9–3.3	<0.0001	1.5	1.1–2.0	0.03	1.6	1.2–2.2	0.005
Hyperlipidemia‡ (yes vs no)		2.0	1.5–2.6	<0.0001	1.5	1.1–2.2	0.02	1.6	1.1–2.2	0.01
HBV coinfection‡ (yes vs no)		1.5	0.9–2.6	0.14						
HCV coinfection‡ (yes vs no)		1.1	0.8–1.6	0.5						
Plasma viral load >50 cp/mL		0.9	0.7–1.3	0.2						
CD4+ Lymphocytes/mm3				<0.0001			<0.0001			<0.0001
	<200 vs >500	2.5	1.6–4.0		2.5	1.5–4.0		2.6	1.7–4.1	
	200–350 vs >500	1.9	1.3–2.8		1.6	1.1–2.4		1.7	1.1–2.5	
	350–500 vs >500	1.3	0.9–1.9		1.2	0.8–1.7		1.2	0.8–1.7	
Delay since HIV diagnosis (years)				0.6						
	5–10 vs <5	0.7	0.5–1.2							
	10–15 vs <5	0.9	0.6–1.3							
	>15 vs <5	0.9	0.6–1.3							
AIDS stage (yes vs no)		2.0	1.5–2.7	<0.0001	1.4	1.0–2.0	0.04	1.5	1.1–2.0	0.02
Baseline creatinine clearance				<0.0001			<0.0001			<0.0001
	60–70 vs >90	19.9	12.7–31.2		15.8	9.4–26.6		15.8	9.4–26.5	
	70–80 vs >90	7.7	4.9–12.2		7.1	4.3–11.7		7.1	4.3–11.8	
	80–90 vs >90	2.0	1.1–3.4		2.0	1.1–3.7		2.1	1.1–3.8	
Exposure to NRTI (ever vs never)		3.5	1.3–9.6	0.01						
Exposure to NNRTI (ever vs never)		1.1	0.9–1.5	0.2						
Exposure to PI		2.0	1.4–2.8	<0.001	1.3	0.8–1.9	0.29			
Exposure to tenofovir (ever vs never)		2.2	1.6–3.0	<0.0001	2.0	1.4–2.8	<0.001			
Exposure to tenofovir				<0.0001						<0.0001
	0–6 months vs never	3.0	1.8–4.9					2.7	1.5–4.7	
	6–12 months with PI vs never	4.1	2.1–8.1					4.3	2.0–9.2	
	6–12 months without PI vs never	1.8	0.9–3.5					1.8	0.9–3.6	
	>12 months with PI vs never	2.7	1.8–3.9					3.0	2.0–4.4	
	>12 months without PI vs never	1.6	1.1–2.6					1.3	0.8–1.9	

Results of univariable and multivariables Poisson regressions.

IRR : Incidence Rate Ratio; HBV: Hepatitis B Virus; HCV: Hepatitis C Virus.

* With exposure to tenofovir as ever/nerver;

† With exposure to tenofovir stratified by exposure to joint PI exposure;

‡ See Methods section for definition.

In the multivariable analysis (Table 2), factors independently associated with a higher incidence of CKD were: older age, female gender, history or presence of diabetes, history or presence of hyperlipidemia, history or presence of high blood pressure, current CD4+ lymphocytes cell count <200/mm^3^, AIDS stage, baseline creatinine clearance <80 ml/mn and exposure to tenofovir (IRR = 2.0; 95% CI [1.4–2.8]). When introduced individually in the final model, none of individual PIs was significantly associated with higher incidence of CKD, data not shown.

In a first additional analysis, taking into account concomitant exposure ≥6 months of PIs and tenofovir, exposure to tenofovir was associated with a higher incidence of CKD when co-administrated with PIs: IRR = 3.0 (95% CI [2.0–4.4]) vs 1.3 [0.9–2.0] without joint PIs (p<0.001), data not shown.

In a second additional analysis, taking into account cumulative exposure to tenofovir, we found that tenofovir exposure >12 months was independently associated with a higher incidence of CKD when concomitant exposure to PI was noticed: IRR = 3.0 (95% CI [2.0–4.4]) vs 1.3[0.9–1.9] without joint PIs (p<0.001). When cumulative exposure to tenofovir was between 6 and 12 months, the difference between risk of CKD, whether or not PIs were concomitantly prescribed, was close to the statistical significance [IRR: 4.3 and 1.8 respectively, p = 0.07].

Among 209 patients who developed CKD, 184 (88.0%) had a baseline eGFR between 60 and 90 ml/min/1.73 m2 and 160 (76.6%) a eGFR between 60 and 80 ml/min/1.73 m2.

All patients developing CKD but four carried at least one non HIV-related risk factors of CKD (female gender, age >45 years, diabetes, hyperlipidemia, baseline MDRD <80 mL/min/1.76 m2), 186 (89.0%) carried at least two of them and 133 (63.6%) at least three. A distribution of these factors stratified by exposure to tenofovir is shown in table 3.

**Table 3:** Distribution of risk factors at the end of follow-up among 209 patients with incident CKD and 4141 without CKD stratified by exposure to tenofovir, ANRS CO3 Aquitaine Cohort 2004–2012.

		Patients with incident CKD N = 209		Patients without incident CKD N = 4141	
Exposure to tenofovir		Never	Ever	Never	Ever
		N = 49 (23.4%)	N = 160 (76.6%)	N = 1033 (25.0%)	N = 3108 (75.0%)
Women		21 (42.9)	60 (37.5)	256 (24.8)	777 (25.0)
Age (years)					
	<45	12 (24.5)	38 (23.7)	445 (43.1)	1260 (40.5)
	45–60	25 (51.0)	82 (51.3)	449 (43.5)	1539 (49.5)
	>60	12 (24.5)	40 (25.0)	139 (13.5)	309 (9.9)
Baseline MDRD (mL/min/1.73 m2)					
	60–70	24 (49.0)	62 (38.8)	97 (9.4)	213 (6.9)
	70–80	17 (34.7)	57 (35.6)	183 (17.7)	511 (16.4)
	80–90	3 (6.1)	21 (13.1)	221 (21.4)	723 (23.3)
	>90	5 (10.2)	20 (12.5)	532 (51.5)	1661 (53.4)
History or presence of diabetes		8 (16.3)	21 (13.1)	58 (5.6)	165 (5.3)
History or presence of hyperlipidemia		34 (69.4)	112 (70.0)	588 (56.9)	1772 (57.0)
Exposure to tenofovir with joint PI ≥6 months		0 (0.0)	72 (34.5)	0 (0.0)	820 (26.4)

Data are n (%).

## Discussion

In a large clinic-based cohort of HIV-infected patients, we report a two fold increase of incidence of CKD when tenofovir was administered in combination with PIs (IRR = 3.1 vs 1.3 in the absence of PIs).

We report an incidence rate of CKD (0.95% PYFU) very close to those already reported in other cohorts; i.e 1.12% PYFU in the John Hopkins HIV Clinical Cohort (using MDRD equation) and 1.05% PYFU in the EuroSIDA Cohort (using Cockcroft-Gault formula) [7], [11]. Among patients with incident CKD, the median annual decline of eGFR was 9.0 ml/mn/1.73 ^2^ which is of clinical significance [27].

As others, we found that traditional risk factors (older age, diabetes, hyperlipidemia and pre-existing mild renal dysfunction) were associated with a higher incidence of CKD [3].

The impact of age reflects the physiological change of the glomerular filtration rate when ageing. With improvement of life expectancy in ART-treated HIV patients, an increasing number of elderly patients is surviving with HIV and a high risk of CKD in patients aged over 60 years can be observed: (IRR = 2.5 when age >60 years compared to age <45 years).

As expected, high blood pressure, diabetes mellitus and hyperlipidemia had a deleterious role on the onset of CKD. Therefore, these characteristics are important to consider in the daily management given their high prevalence in patients receiving ART and their modifiable nature. The potential confounding role of PIs in the associations of metabolic disorders with higher incidence of CKD was taken into account by maintaining the inclusion of the use of PIs in the final multivariate model.

More constantly than in HIV-negative population, women have a higher incidence of CKD among HIV-infected individuals [7], [11], [20]. Physiological differences between men and women may explain such effect but we cannot exclude that the adjustment of the simplified MDRD equation in females (i.e×0.742), which takes into account a lower relative muscle mass among women compared to men, yield an overestimation of MDRD. Indeed, differences in muscle mass between men and women may be lower in HIV infected population because of their frequent sarcopenia [28].

Specific HIV-related factor independently associated with a higher risk of CKD in our study were, as reported by other reports, history of AIDS [14], [23] and a low current CD4 count [11], [14] The specific nephrotoxicity of AIDS-defining diseases treatments or prophylaxis (such as the use of trimethoprime-sulfamethoxazole) could not be assessed by lack of reliable data. We chose to include current CD4 count rather than CD4 nadir as the latter is often of unknown value when case management of patients started prior to their enrolment in the ANRS CO3 Aquitaine cohort: we acknowledge that CD4 nadir could have better revealed consequences associated with AIDS defining conditions, which was nevertheless assessed in our study by taking into account the history of AIDS stage.

In accordance with other cohort studies showing a deleterious impact of tenofovir on renal function [11], [17], [20], [22], [29]–[31] we found, as published by Mocroft et al [11] and more recently by Scherzer et al. [32] a significant association between exposure to tenofovir and higher incidence of CKD. This result does not contradict data from several clinical trials assessing the renal safety of tenofovir as, in trials, patients have usually higher baseline eGFR than in observational cohorts like ours where patients are unselected, older with more risk factors such as hypertension, diabetes and dyslipidemia [33], [34]. Tenofovir may be associated with glomerular and above all proximal tubular dysfunction including development of Fanconi syndrome [35]. In a cross sectional analysis performed in the same cohort, cumulative exposure to tenofovir was associated with an increased risk of proximal renal tubular function whether treatment was ongoing or had been discontinued, leading to concern about the reversibility of the phenomenon [36]. If many factors could influence the risk of developing tubular dysfunction, Rodriguez-Novoa et al demonstrated an association between this dysfunction and the homozygosity for the C allele at position −24 of the *ABCC2* gene encoding drug transporters [37] but this association was not confirmed by several other investigations.

Conversely to the Euro-SIDA group study cohort [11], we did not find an independent role of the protease inhibitors as a class in our multivariable analysis. We found a significant impact of exposure to ritonavir-boosted Protease Inhibitors either as a whole or for some individual PI (indinavir, atazanavir and a tendency for lopinavir) on the incidence of CKD, but only in the un-adjusted analysis. Among PIs, Scherzer et al. mentioned an increased risk of CKD only with indinavir [32] but Mocroft et al showed that a longer cumulative exposure to indinavir/r, atazanavir/r or lopinavir/r was associated with a higher rate of CKD. Nevertheless we observed that risk of CKD was higher when TDF was taken concomitantly (during at least 6 months) with PIs than when taken without concomitantly PIs, as relative risks of progression to CKD were respectively 3.0 and 1.3. This result can be compared to the report by Kalayjan et al. [23] who found that tenofovir plus PI/r was associated with a higher risk of CKD (Hazard Odds Ratio = 3.35 [95% CI, 1.40–8,02]). Moreover, when cumulative exposure to tenofovir was taken into account, we found that a concomitant tenofovir and PIs intake was associated with a higher risk of CKD than exposure to tenofovir without PIs mainly when cumulative exposure to tenofovir was >12 months. These findings are consistent with those of other cohort studies showing that patients receiving tenofovir in combination with PI/r had an increase decline in renal function compared with those receiving tenofovir and NNRTIs [17]–[22]. These results may have significant clinical impact in the case management of tenofovir-treated patients as limiting or avoiding concomitant use of PI/r might be important, mainly among those having other risk factors of progressing to CKD, as in our study, no patient developed CKD when exposure to tenofovir was the only risk factor of CKD. PIs studied in our report were mainly the following boosted PIs: atazanavir, lopinavir, fosamprenavir and saquinavir. We cannot assert that our findings could be extrapolated to unboosted PIs or more recently available drugs such as darunavir. The interaction between TDF and PIs on nephrotoxicity in HIV-infected patients should be confirmed in independent larger databases also in order to examine whether this effect is homogeneous or not among different PIs.

In few reports, where drugs were analysed individually, zidovudine, didanosine and efavirenz were associated with increased incidence of CKD [20], [32]. In our report, where ART were studied as a class, we did not find a deleterious impact of exposure to NRTIs and NNRTIs.

Conversely to a recent report [38], we did not find any association between HCV co-infection and progression to CKD, but we were not able to distinguish patients with active HCV infection as in the Eurosida Study.

The susceptibility to renal impairment of black patients, considered as especially susceptible to HIVAN, could not be evaluated in our study as ethnicity is not registered in our database, where patients are mostly (>90%) white.

As in the Euro SIDA cohort, progressing to CKD was very infrequent in our study when the estimated GFR at baseline was above 90 mL/min/1.73 m2. The vast majority of patients who developed CKD (77%) had a baseline estimated renal clearance between 60 and 80 mL/min/1.73 m2. This level of eGFR appeared to be the main contributing risk of the occurrence of CKD. This result seems to us an important message that remains somewhat under-appreciated by clinicians while it should have implications for closer monitoring and specific care management of HIV infected individuals. It seems moreover interesting to notice that we found a relatively similar incidence of CKD during each year of follow-up leading to a regular increase of the cumulative risk over time. This evolution suggests that the monitoring of the glomerular filtration rate needs to be regularly performed throughout the follow-up regardless of the level of initial measurement.

Presence of proteinuria, identifying early kidney renal dysfunction, was not assessed in our study. Although no study had examined the usefulness of its systematic screening in HIV infected individuals, there is some evidence that early recognition of chronic renal impairment might be beneficial [39]–[41].

We know that the MDRD equation was developed in people with CKD, and, as such, that its major limitations are imprecision and systematic underestimation of measured GFR at higher levels [25]. As these limitations are present among patients taking tenofovir whether or not concomitant PIs intake, we think that using MDRD formula may be associated with a non differential measurement bias, leading statistical association measurements (RR, IRR) to null value (i.e. 1). Finding IRR >1 actually reinforce our significant results.

In conclusion, incidence of CKD occurs frequently among treated HIV-infected patients and, with ageing of HIV-infected population, might represent an important issue in terms of mortality and incidence of cardiovascular diseases in the next future. Regular assessment of renal function and evaluation of other CKD risk factors are warranted in HIV-infected patients from the time of HIV diagnosis [40] and should be taken into account in the choices of ART. Our results provide evidence that, in patients with eGFRs between 60 and 80 ml/mn/1.73 m2, a thorough control of CKD risk factors (mainly high blood pressure, diabetes and hyperlipidemia) is warranted and that use of TDF, especially when co-administered with PIs, should be mentioned as a relative contraindication in presence of additional risk factors of CKD.

## References

[1] K/DOQI clinical practice guidelines for chronic kidney disease: evaluation, classification, and stratification. Am J Kidney Dis 39: S1–266. 11904577

[2] Di Angelantonio E, Danesh J, Eiriksdottir G, Gudnason V (2007) Renal function and risk of coronary heart disease in general populations: new prospective study and systematic review. PLoS Med 4: e270 doi:10.1371/journal.pmed.0040270 17803353 10.1371/journal.pmed.0040270 PMC1961630

[3] Winston J, Deray G, Hawkins T, Szczech L, Wyatt C, et al (2008) Kidney disease in patients with HIV infection and AIDS. Clin Infect Dis 47: 1449–1457 doi:10.1086/593099 18947327 10.1086/593099

[4] Bigé N, Lanternier F, Viard J-P, Kamgang P, Daugas E, et al (2012) Presentation of HIV-associated nephropathy and outcome in HAART-treated patients. Nephrol Dial Transplant 27: 1114–1121 doi:10.1093/ndt/gfr376 21745806 10.1093/ndt/gfr376

[5] Deti EK, Thiebaut R, Bonnet F, Lawson Ayayi S, Dupon M, et al (2010) Prevalence and factors associated with renal impairment in HIV-infected patients, ANRS C03 Aquitaine Cohort, France. HIV Med 11: 308–317. 20002500 10.1111/j.1468-1293.2009.00780.x

[6] Mocroft A, Kirk O, Gatell J, Reiss P, Gargalianos P, et al (2007) Chronic renal failure among HIV-1-infected patients. AIDS 21: 1119–1127 doi:10.1097/QAD.0b013e3280f774ee 17502722 10.1097/QAD.0b013e3280f774ee

[7] Lucas GM, Lau B, Atta MG, Fine DM, Keruly J, et al (2008) Chronic kidney disease incidence, and progression to end-stage renal disease, in HIV-infected individuals: a tale of two races. J Infect Dis 197: 1548–1557 doi:10.1086/587994 18422458 10.1086/587994 PMC2553209

[8] Sorlí ML, Guelar A, Montero M, González A, Rodriguez E, et al (2008) Chronic kidney disease prevalence and risk factors among HIV-infected patients. J Acquir Immune Defic Syndr 48: 506–508 doi:10.1097/QAI.0b013e31817bbecb 18614921 10.1097/QAI.0b013e31817bbecb

[9] Levey AS, Eckardt K-U, Tsukamoto Y, Levin A, Coresh J, et al (2005) Definition and classification of chronic kidney disease: a position statement from Kidney Disease: Improving Global Outcomes (KDIGO). Kidney Int 67: 2089–2100 doi:–10.1111/j.1523–1755.2005.00365.x 15882252 10.1111/j.1523-1755.2005.00365.x

[10] Prevalence of chronic kidney disease and associated risk factors–United States, 1999–2004 (2007) MMWR Morb Mortal Wkly Rep. 56: 161–165. 17332726

[11] Mocroft A, Kirk O, Reiss P, De Wit S, Sedlacek D, et al (2010) Estimated glomerular filtration rate, chronic kidney disease and antiretroviral drug use in HIV-positive patients. AIDS 24: 1667–1678 doi:10.1097/QAD.0b013e328339fe53 20523203 10.1097/QAD.0b013e328339fe53

[12] Szczech LA, Gange SJ, van der Horst C, Bartlett JA, Young M, et al (2002) Predictors of proteinuria and renal failure among women with HIV infection. Kidney Int 61: 195–202 doi:10.1046/j.1523-1755.2002.00094.x 11786101 10.1046/j.1523-1755.2002.00094.x

[13] Krawczyk CS, Holmberg SD, Moorman AC, Gardner LI, McGwin G Jr (2004) Factors associated with chronic renal failure in HIV-infected ambulatory patients. AIDS 18: 2171–2178. 15577650 10.1097/00002030-200411050-00009

[14] Leport C, Bouteloup V, Rossert J, Garre M, Iordache L, et al (2009) Long-term evolution and determinants of renal function in HIV-infected patients who began receiving combination antiretroviral therapy in 1997–1999, ANRS CO8 APROCO-COPILOTE. Clin Infect Dis 49: 1950–1954 doi:10.1086/648445 19911986 10.1086/648445

[15] Kearney BP, Mathias A, Mittan A, Sayre J, Ebrahimi R, et al (2006) Pharmacokinetics and safety of tenofovir disoproxil fumarate on coadministration with lopinavir/ritonavir. J Acquir Immune Defic Syndr 43: 278–283 doi:10.1097/01.qai.0000243103.03265.2b 17079992 10.1097/01.qai.0000243103.03265.2b

[16] Kiser J, Carten M, Aquilante C, Anderson P, Wolfe P, et al (2007) The Effect of Lopinavir//Ritonavir on the Renal Clearance of Tenofovir in HIV-infected Patients. Clin Pharmacol Ther 83: 265–272. 17597712 10.1038/sj.clpt.6100269

[17] Fux CA, Christen A, Zgraggen S, Mohaupt MG, Furrer H (2007) Effect of tenofovir on renal glomerular and tubular function. AIDS 21: 1483–1485 doi:[]10.1097/QAD.0b013e328216f15b [doi] 17589197 10.1097/QAD.0b013e328216f15b

[18] Goicoechea M, Liu S, Best B, Sun S, Jain S, et al (2008) Greater tenofovir-associated renal function decline with protease inhibitor-based versus nonnucleoside reverse-transcriptase inhibitor-based therapy. J Infect Dis 197: 102–108 doi:10.1086/524061 18171292 10.1086/524061

[19] Horberg M, Tang B, Towner W, Silverberg M, Bersoff-Matcha S, et al (2010) Impact of tenofovir on renal function in HIV-infected, antiretroviral-naive patients. J Acquir Immune Defic Syndr 53: 62–69 doi:10.1097/QAI.0b013e3181be6be2 19838127 10.1097/QAI.0b013e3181be6be2

[20] Tordato F, Cozzi Lepri A, Cicconi P, De Luca A, Antinori A, et al (2011) Evaluation of glomerular filtration rate in HIV-1-infected patients before and after combined antiretroviral therapy exposure. HIV Med 12: 4–13 doi:–10.1111/j.1468–1293.2010.00855.x 20584091 10.1111/j.1468-1293.2010.00855.x

[21] Albini L, Cesana BM, Motta D, Focà E, Gotti D, et al (2012) A randomized, pilot trial to evaluate glomerular filtration rate by creatinine or cystatin C in naive HIV-infected patients after tenofovir/emtricitabine in combination with atazanavir/ritonavir or efavirenz. J Acquir Immune Defic Syndr 59: 18–30 doi:10.1097/QAI.0b013e31823a6124 21992924 10.1097/QAI.0b013e31823a6124

[22] Young J, Schäfer J, Fux CA, Furrer H, Bernasconi E, et al (2012) Renal function in patients with HIV starting therapy with tenofovir and either efavirenz, lopinavir or atazanavir. AIDS 26: 567–575 doi:10.1097/QAD.0b013e32834f337c 22398568 10.1097/QAD.0b013e32834f337c

[23] Kalayjian RC, Lau B, Mechekano RN, Crane HM, Rodriguez B, et al.. (2012) Risk factors for chronic kidney disease in a large cohort of HIV-1 infected individuals initiating antiretroviral therapy in routine care. AIDS (London, England). doi:10.1097/QAD.0b013e328357f5ed 10.1097/QAD.0b013e328357f5ed PMC3531628 22824630

[24] Thiébaut R, Morlat P, Jacqmin-Gadda H, Neau D, Mercié P, et al (2000) Clinical progression of HIV-1 infection according to the viral response during the first year of antiretroviral treatment. Groupe d’Epidémiologie du SIDA en Aquitaine (GECSA). AIDS 14: 971–978. 10853978 10.1097/00002030-200005260-00008

[25] Levey AS, Coresh J, Greene T, Marsh J, Stevens LA, et al (2007) Expressing the Modification of Diet in Renal Disease Study equation for estimating glomerular filtration rate with standardized serum creatinine values. Clin Chem 53: 766–772 doi:10.1373/clinchem.2006.077180 17332152 10.1373/clinchem.2006.077180

[26] Levey AS, Stevens LA, Schmid CH, Zhang YL, Castro AF 3rd, et al (2009) A new equation to estimate glomerular filtration rate. Ann Intern Med 150: 604–612. 19414839 10.7326/0003-4819-150-9-200905050-00006 PMC2763564

[27] Jones C, Roderick P, Harris S, Rogerson M (2006) Decline in kidney function before and after nephrology referral and the effect on survival in moderate to advanced chronic kidney disease. Nephrol Dial Transplant 21: 2133–2143 doi:10.1093/ndt/gfl198 16644779 10.1093/ndt/gfl198

[28] Buehring B, Kirchner E, Sun Z, Calabrese L (2011) The Frequency of Low Muscle Mass and Its Overlap With Low Bone Mineral Density and Lipodystrophy in Individuals With HIV-A Pilot Study Using DXA Total Body Composition Analysis. Journal of Clinical Densitometry. doi:10.1016/j.jocd.2011.10.003 10.1016/j.jocd.2011.10.003 22169198

[29] Campbell LJ, Ibrahim F, Fisher M, Holt SG, Hendry BM, et al (2009) Spectrum of chronic kidney disease in HIV-infected patients. HIV Med 10: 329–336 doi:–10.1111/j.1468–1293.2008.00691.x 19226409 10.1111/j.1468-1293.2008.00691.x

[30] De Beaudrap P, Diallo MB, Landman R, Guèye NF, Ndiaye I, et al (2010) Changes in the renal function after tenofovir-containing antiretroviral therapy initiation in a Senegalese cohort (ANRS 1215). AIDS Res Hum Retroviruses 26: 1221–1227 doi:10.1089/aid.2009.0261 20854202 10.1089/aid.2009.0261

[31] Brennan A, Evans D, Maskew M, Naicker S, Ive P, et al (2011) Relationship between renal dysfunction, nephrotoxicity and death among HIV adults on tenofovir. AIDS 25: 1603–1609 doi:10.1097/QAD.0b013e32834957da 21646902 10.1097/QAD.0b013e32834957da PMC3586413

[32] Scherzer R, Estrella M, Li Y, Choi AI, Deeks SG, et al (2012) Association of tenofovir exposure with kidney disease risk in HIV infection. AIDS 26: 867–875 doi:10.1097/QAD.0b013e328351f68f 22313955 10.1097/QAD.0b013e328351f68f PMC3736566

[33] Cooper RD, Wiebe N, Smith N, Keiser P, Naicker S, et al (2010) Systematic review and meta-analysis: renal safety of tenofovir disoproxil fumarate in HIV-infected patients. Clin Infect Dis 51: 496–505 doi:10.1086/655681 20673002 10.1086/655681

[34] Gallant JE, Winston JA, DeJesus E, Pozniak AL, Chen SS, et al (2008) The 3-year renal safety of a tenofovir disoproxil fumarate vs. a thymidine analogue-containing regimen in antiretroviral-naive patients. AIDS 22: 2155–2163 doi:[]10.1097/QAD.0b013e3283112b8e [doi] 18832879 10.1097/QAD.0b013e3283112b8e

[35] Gupta SK (2008) Tenofovir-associated Fanconi syndrome: review of the FDA adverse event reporting system. AIDS Patient Care STDS 22: 99–103 doi:10.1089/apc.2007.0052 18260800 10.1089/apc.2007.0052

[36] Dauchy FA, Lawson-Ayayi S, de La Faille R, Bonnet F, Rigothier C, et al (2011) Increased risk of abnormal proximal renal tubular function with HIV infection and antiretroviral therapy. Kidney Int 80: 302–309 doi:[]10.1038/ki.2011.124 [doi] 21544066 10.1038/ki.2011.124

[37] Rodriguez-Novoa S, Labarga P, Soriano V, Egan D, Albalater M, et al (2009) Predictors of kidney tubular dysfunction in HIV-infected patients treated with tenofovir: a pharmacogenetic study. Clin Infect Dis 48: e108–16 doi:10.1086/598507 19400747 10.1086/598507

[38] Peters L, Grint D, Lundgren JD, Rockstroh JK, Soriano V, et al (2012) Hepatitis C virus viremia increases the incidence of chronic kidney disease in HIV-infected patients. AIDS 26: 1917–1926 doi:10.1097/QAD.0b013e3283574e71 22781222 10.1097/QAD.0b013e3283574e71

[39] Gerstein HC, Mann JFE, Yi Q, Zinman B, Dinneen SF, et al (2001) Albuminuria and Risk of Cardiovascular Events, Death, and Heart Failure in Diabetic and Nondiabetic Individuals. JAMA 286: 421–6 doi:10.1001/jama.286.4.421 11466120 10.1001/jama.286.4.421

[40] Gupta SK (2005) Tenofovir and changes in renal function. Clin Infect Dis 41: 570–1; author reply 571. doi:CID36718 [pii] 10.1086/432124. 10.1086/432124 16028175

[41] Clark WF, Macnab JJ, Sontrop JM, Jain AK, Moist L, et al (2011) Dipstick proteinuria as a screening strategy to identify rapid renal decline. J Am Soc Nephrol 22: 1729–1736 doi:10.1681/ASN.2010111217 21807890 10.1681/ASN.2010111217 PMC3171943

